# Connexin45 Is Expressed in Vascular Smooth Muscle but Its Function Remains Elusive

**DOI:** 10.1371/journal.pone.0042287

**Published:** 2012-07-27

**Authors:** Volker J. Schmidt, Alexander Jobs, Julia von Maltzahn, Philipp Wörsdörfer, Klaus Willecke, Cor de Wit

**Affiliations:** 1 Institut für Physiologie, Universität zu Lübeck, Lübeck, Germany; 2 Institut für Genetik, Universität Bonn, Bonn, Germany; 3 Plastisch- und Handchirurgische Klinik, Universitätsklinikum Erlangen, Erlangen, Germany; University of Colorado Denver, United States of America

## Abstract

Connexins (Cx) form gap junctions and allow the coordination of cellular behaviour. In vessels, expression of Cx40, Cx37, and Cx43 is well established and specifically Cx40 serves important functions in endothelial cells. In contrast, expression and physiological functions of Cx45 is unclear although its expression has been suggested in vascular smooth muscle (VSM). Therefore, we studied expression and function of Cx45 in vessels using different mice models allowing to identify and delete Cx45. Smooth muscle cell (SMC)-specific deletion was achieved by the Cre/loxP system using Cre-recombinase driven by a Nestin promoter. Deletion of Cx45 leads concomitantly to the expression of enhanced green fluorescence protein (EGFP) in these mice. Conduction of vasomotor responses was studied in cremasteric arterioles using intravital microscopy and arterial pressure was measured telemetrically. Cx45 is transcriptionally expressed in VSM as detected by EGFP expression in SMC-specific Cx45-deficient mice (Cx45fl/fl:Nestin-Cre) but not in endothelial cells (Cx45fl/fl:TIE2-Cre). Moreover, EGFP was located at VSM cell borders in arterioles of transgenic mice carrying an EGFP-tagged Cx45. Expectedly, arteriolar conduction of dilations evoked by the endothelium-dependent agonist acetylcholine were not different between Cx45fl/fl:Nestin-Cre mice and controls carrying homozygously a floxed Cx45 gene (Cx45fl/fl). Surprisingly, the amplitude of locally initiated endothelium-independent constrictions (K^+^) and dilations (adenosine) declined similarly with distance in both genotypes indicating an intact VSM conduction pathway also in mice being deficient for Cx45 in VSM. Arterial pressure was not different between freely moving Cx45fl/fl and Cx45fl/fl:Nestin-Cre mice during day or night. We conclude that Cx45 is physiologically expressed in VSM, but not in EC in murine arterioles. However, Cx45 is dispensable for the conduction of vasomotor responses along these arterioles. Possibly, other Cx functionally replace the lack of Cx45 in VSM. The reported role of Cx45 in renin secretion does not seem to alter arterial pressure in freely moving mice.

## Introduction

Gap junctions enable fast signalling between adjacent cells und thus connect individual cells into a functional unit or syncitium. Such signalling is also pivotal in vessels in the microcirculation since thereby vasomotor signals may communicate along the vascular wall, which lead to the coordination of vascular cell behaviour and synchronous vasoconstriction or vasodilation. Arterioles in microcirculatory networks cover substantial distances and thus only concordant diameter changes along their overall length efficiently modulate vascular resistance thereby adjusting organ perfusion to its demand in a wide dynamic range. The functional coupling can be investigated by brief locally confined application of certain vasoactive compounds to elicit a local vasomotor response. The response is not confined to the site of stimulation but travels to remote up- and downstream sites without measurable time delay and is therefore termed conducted response [1]–[3]. The high conduction velocity conformed by the measurement of remote membrane potential changes led to the conclusion that electrotonic transmission of a locally initiated membrane potential change acts as the major coordinating signal along the arterioles [4], [5].

Gap junction channels are composed of connexin (Cx) protein subunits that provide conduits of low electrical resistance between cells [6]. The connexin gene family comprises 20 members in the mouse genome which are named according to the molecular weight of the protein [7]. Of these isoforms, four Cx are expressed in vascular tissues, namely Cx37, Cx40, Cx43, and Cx45 [8]–[11] which exhibit distinct gating properties and conductivities [12]–[15]. The coexistence of various isoforms suggests that distinct Cx serve specific functions which are, however, difficult to unravel because a specific and effective pharmacologic blockade is not readily available. However, genetically altered mice being ubiquitously deficient for Cx have been shown promising to dissect specific Cx functions [16]–[20]. In mice deficient for Cx40, which is expressed abundantly in endothelial cells, the conduction of vasodilations along arterioles in the microcirculation initiated by endothelium-dependent dilators is impaired suggesting the requirement of Cx40 to support the transmission of vasodilator signals along the endothelial cell layer [21]–[23]. In contrast, Cx37-deficient mice did not exhibit such a defect despite the fact that Cx37 is likewise abundantly expressed in endothelial cells [24]. Interestingly, the conduction of dilations initiated by substances acting independently of the endothelium as well as the conduction of vasoconstrictions initiated by local depolarizations are unaffected by the lack of Cx40. This suggests that, in addition to a pathway along the endothelial cell layer, a secondary conduction pathway may be provided by the smooth muscle layer in which Cx40 is not a crucial component [25].

Cx43 and Cx45 are suggestedly expressed in vascular smooth cells of cerebral arteries, the aorta and other large conducting arteries [9], [26]. These conclusions were mostly derived from immunostaining relying on the specificity of antibodies to detect the protein of interest. Functional data are not available partially due to the fact that animals with ubiquitous deletion of Cx45 are not viable [27], [28]. Thus, vascular expression of Cx45 and particularly its function is unclear. In the kidney, Cx45 was postulated to be expressed in smooth muscle cells of afferent and efferent arterioles and to be involved in the regulation of renin secretion [29]. Its lack in smooth muscle cells resulted in elevated arterial pressure [29]. However, its expression and function in the overall circulation as well as its functional role in conducted responses in arterioles remains unknown.

We hypothesized that Cx45 is expressed in vascular smooth muscle throughout the circulation, connects smooth muscle cells homocellularly, and is required to support the conduction of vasomotor responses along the smooth muscle layer. To test these hypotheses, we examined Cx45 expression using mice carrying a Cx45 tagged with enhanced green fluorescence protein (EGFP) [30]. Furthermore, Cx45 function was analysed by studying conducted responses in animals deficient for Cx45 specifically in smooth muscle cells using the Cre/loxP-System in which the Cre-recombinase was driven by the promoter of the filament protein nestin. In these latter animals, Cx45 deletion and accordingly its expression in wildtype animals were verified by the expression of EGFP in case of successful removal of the Cx45 gene [31], [32]. Similarly, possible Cx45 expression was examined in mice carrying a Cre-recombinase driven by the endothelial-cell specific promoter TIE2.

## Materials and Methods

### Mice

The investigation conformed to the guide for the care and use of laboratory animals published by the US National Institutes of Health, was performed in accordance with the German law for animal welfare and was approved by local authorities (Landwirtschafts- und Umweltministerium Schleswig-Holstein, V312-72241.122-2). All surgery was performed under anesthesia (see below), and all efforts were made to minimize suffering. Cell-specific deletion of Cx45 was achieved using mice that harbor a floxed Cx45 gene (Cx45fl). The deletion of Cx45 accomplished by Cre-recombinase activity leads concomitantly to the expression of the reporter enhanced green fluorescent protein (EGFP) [31], [32]. Cre-recombinase expression was controlled by the promoter of the intermediate filament Nestin (Nestin-Cre) which is active in smooth muscle cells during development [33], [34] or by the endothelial-cell specific promoter TIE2 [35]. Littermates homozygous for the floxed gene without Cre-recombinase served as controls (Cx45fl/fl). In addition, mice carrying an EGFP-tagged Cx45 [30] were studied in immunostaining and their littermates without EGFP-tagged Cx45 were used as controls. Mice were genotyped by PCR from tail-tip biopsies as described [30], [31].

### Experimental setup

Anesthesia was induced by intraperitoneal injection of midazolam (5 mg/kg), fentanyl (0.05 mg/kg), and medetomidine (0.5 mg/kg) followed by continuous infusion (jugular vein). The animals were intubated by tracheotomy to ensure airway patency and their esophageal temperature maintained at 37°C. The cremaster muscle was prepared for intravital microscopy as described [36] and superfused with bicarbonate-buffered (pH 7.4, 34°C) salt solution (in mmol/L: Na^+^ 143, K^+^ 5, Ca^2+^ 2.5, Mg^2+^ 1.2, Cl^−^ 128, HCO_3_^−^ 25, SO_4_^2−^ 1.2, and H_2_PO_4_^−^ 1.2) gassed with 5% CO_2_ and 95% N_2_ (pO_2_∼30 and pCO_2_∼40 mm Hg). The microcirculation was studied using a microscope (Axioskop FS, ZEISS, Göttingen, Germany) mounted on a movable platform and equipped with a charge-coupled video camera. One to three second-order arterioles in the central region of the tissue were studied in each animal. Microscopic images were displayed on a monitor (700-fold magnification) and recorded on videotape for later measurement of luminal diameter after digitization (resolution after digitization ∼1 µm).

### Experimental protocols

Conduction of vasomotor responses was studied by focally confined stimulation using a glass micropipette (tip 1–2 µm) placed in close proximity to the arteriolar wall through which vasoactive compounds (acetylcholine [ACh] 1 mmol/L, adenosine 10 mmol/L, or KCl 3 mol/L) were ejected by a short pressure pulse (140 kPa, 200–700 ms) [25]. If a response was recorded at the stimulation site, the same stimulation was repeated while observing remote, upstream sites in a distance of 300, 600, 900, or 1200 µm. Each substance was investigated in duplicate at a single vessel before studying either the next substance or a second vessel. In all animals, the genotype was confirmed by checking for EGFP expression in the microcirculation using fluorescence microscopy. At the end of the experiment maximal vessel diameter was determined by combined superfusion of sodium nitroprusside, ACh, and adenosine (each 30 µmol/L) before the animal was killed by pentobarbital.

### Measurement of arterial pressure by radiotelemetry

In separate animals, arterial pressure was measured by radiotelemetry. Mice were anesthetized using isoflurane (1 to 1.5%) and surgically implanted with microminiaturized radiotelemeters (TA11PA-C10, Data Sciences International, St. Paul, MN, USA). The catheter was introduced into the left carotid artery and advanced to the aortic arch as described [37]. After stabilization and recovery (5 d) arterial pressure was recorded every five minutes for two minutes during several days (day 5 to 7 and day 11 to 14 after implantation) at 500 Hz using commercially available software (Data Sciences International). Heart rate was determined offline from the pressure curve.

### Immunostaining

For whole-mount immunolabeling mice were anesthetized, the cremaster muscle prepared, removed shortly after killing the animal, and pinned flat in dishes covered with silicone (Sylgard, Dow Corning). Other tissues (aorta, femoral, carotid and gracilis artery, mesenterium) were harvested afterwards [38]. In animals expressing the reporter EGFP instead of Cx45 and their respective controls tissues were briefly incubated with nuclear stain (TO-PRO-3 or DAPI, Invitrogen) without fixation and after rigorous washing immediately studied using a confocal laser-scanning microscope and appropriate filter wheels (Leica TCS). Tissues obtained from EGFP-tagged Cx45 mice and their controls were further processed by fixation (4.5% formaldehyde for 5 min) and washing (phosphate buffered saline, PBS) before being blocked and permeabilized (1% BSA, 0.2% TritonX-100 in PBS, 2 h). The primary antibody (anti-Cx40 1∶400, Millipore or anti-Cx37 1∶400, Alpha Diagnostics) was added in blocking solution overnight at 4°C. After washing (1% TritonX-100 in PBS, 1 h; PBS, 30 min) immuncomplexes were visualized using goat anti-rabbit IgG (1∶800; Alexa Fluor 594, Molecular Probes) and nuclei were stained with TO-PRO-3 [39], [40]. To enhance visualization of EGFP in EGFP-tagged Cx45 mice an appropriate antibody (anti-GFP coupled to Alexa Fluor 488, Molecular Probes) was applied for 4 h. The tissue was again washed several times, embedded in Mowiol, and mounted flat on a slide. Staining was visualized using a confocal laser-scanning-microscope and appropriate filters (Leica TCS).

### Statistics and calculations

Vessel diameter changes were normalized to the maximal possible response:


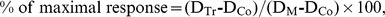


where D_Tr_ is the diameter after treatment, D_Co_ the control diameter before treatment, and D_M_ the maximal possible diameter that is the maximally dilated diameter for dilations or the minimal luminal diameter (zero) for constrictions. To characterize the temporal characteristics of the responses the ‘time to peak’ (interval between stimulus application and attainment of maximal peak) and the ‘response duration’ (interval between stimulus and recovery to resting diameter) were calculated. Comparisons within groups were performed using paired *t* tests, and corrected according to Bonferroni. Data between groups were compared with ANOVA, followed by post hoc analysis of the means. Data are presented as mean±SEM. Differences were considered significant at a corrected error probability of *P*<0.05.

## Results

### Expession of Cx45 in arteries and arterioles

**Figure 1:**
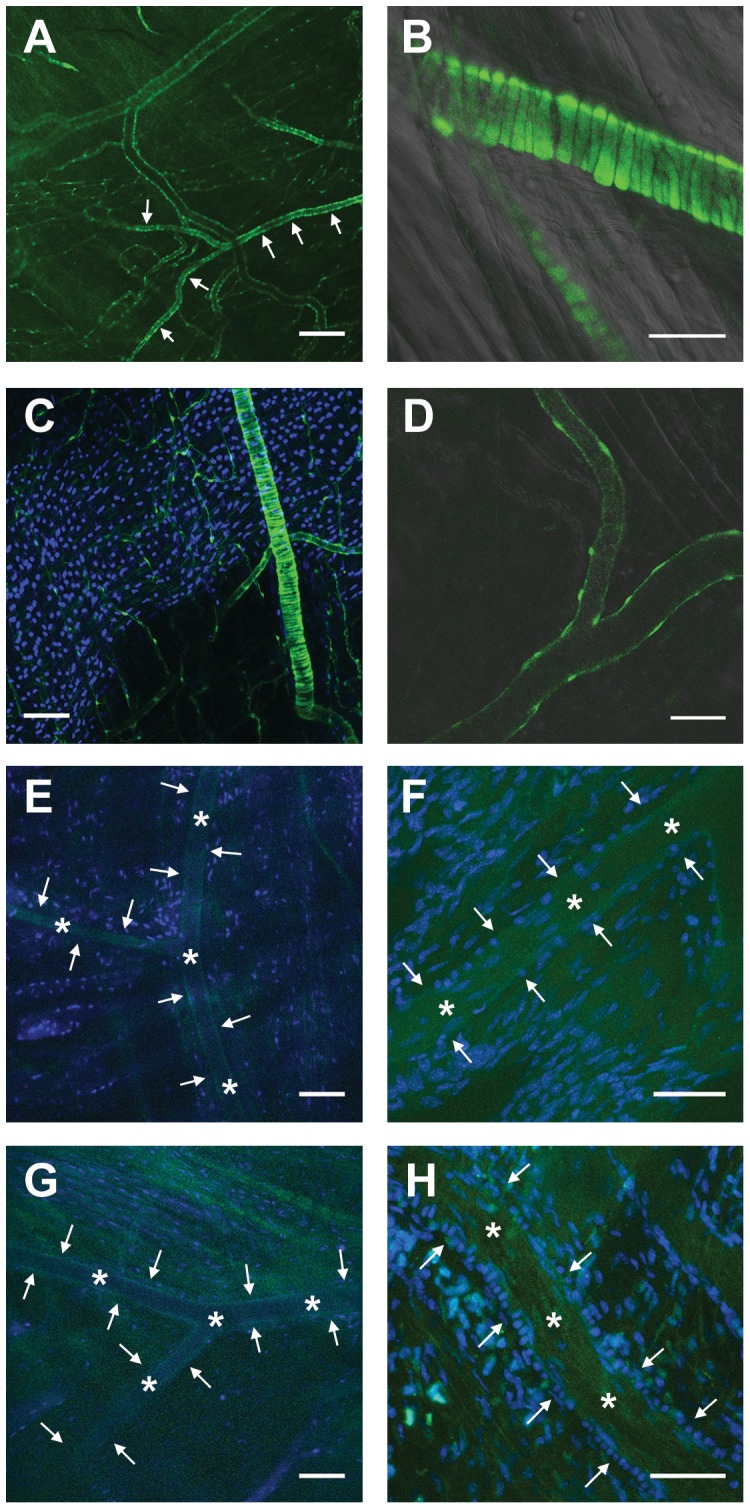
Connexin45 expression in the microcirculation. In situ expression of the reporter enhanced green fluorescent protein (EGFP) in mice harboring a floxed Cx45 gene which deletion by Cre-recombinase activity leads concomitantly to the expression of the reporter. Smooth muscle cell-specific activation of Cre-recombinase was achieved by the Nestin promoter and endothelial-cell specific activation by the TIE2 promoter. Vascular smooth muscle cells are stained in arterioles (arrows in A) as well as in venules, and most likely pericytes in capillaries in the microcirculation of a skeletal muscle (cremaster muscle) in Cx45fl/fl:Nestin-Cre mice. In arterioles, smooth muscle cells can be identified by their typical wrapping around the vessel (B, C). Staining was less bright in venules (D). EGFP was not identified in mice carrying TIE2-controlled Cre-recombinase that were homo- or heterozygous for the Cx45-floxed gene (E and F). Staining was also absent in Cx45fl/fl mice devoid of Cre-recombinase (G and H). Arteriolar lumen is marked (*) and vessel borders are shown by arrows in E to H, nuclei stained in blue (TO-PRO-3) except in A, B, and D. Images are representative for at least n = 3 each genotype. Calibration bars are 200 (A), 100 (C,E,G), or 50 µm (B,D,F,H).

We used two different mouse models to verify the expression of Cx45 in vascular cells and to circumvent problems associated with antibody specificity. The first model consisted of animals which harbor a floxed Cx45 gene (Cx45fl). In these mice, cell-specific deletion of Cx45 accomplished by Cre-recombinase activity leads concomitantly to the expression of the reporter enhanced green fluorescent protein (EGFP) [31], [32]. Cre-recombinase expression was controlled by the promoter of the intermediate filament Nestin. Animals being homozygous for Cx45fl and expressing Nestin-Cre (Cx45fl/fl:Nestin-Cre) were viable and arteriolar vascular smooth muscle cells in the microcirculation were distinctly labelled with EGFP (Fig. 1). Venules exhibited also EGFP staining in the outer layer (Fig. 1D), which was not as bright as staining in arterioles (Fig. 1A, 1C), most likely representing the smaller size of venular smooth muscle cells. Furthermore, EGFP was expressed in cells within or adjacent to capillaries which may represent pericytes (Fig. 1A, 1C). Animals carrying a TIE2-driven Cre-recombinase and being either homo- or heterozygous for Cx45fl (Cx45fl/fl:TIE2-Cre or Cx45fl/wt:TIE2-Cre) were also viable but did not exhibit EGFP staining (Fig. 1E, 1F). Staining was also completely absent in Cx45fl/fl control mice (Fig. 1G, 1H).

**Figure 2:**
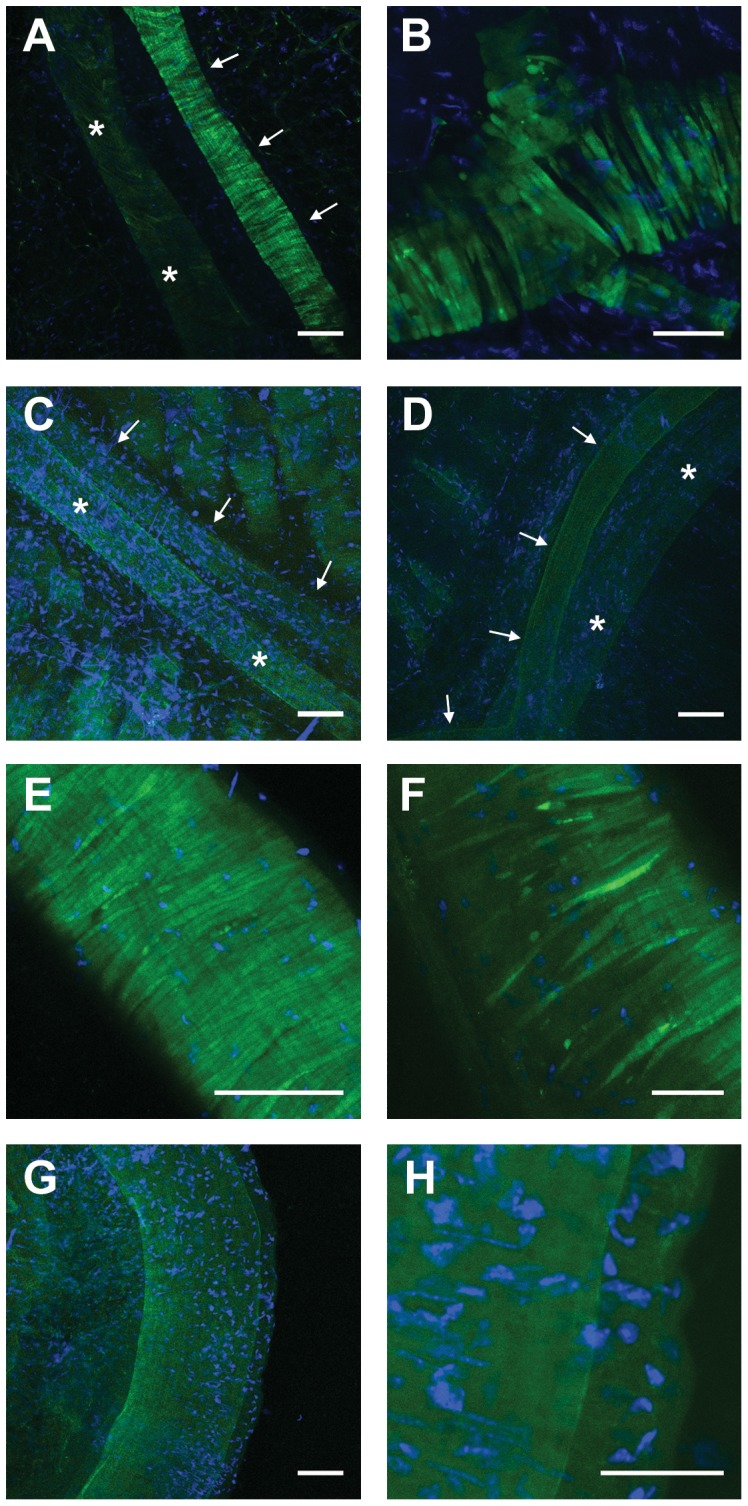
Connexin45 expression in larger vessels. Expression of EGFP in smooth muscle cells of the gracilis artey (A, B) and the femoral artery (E, F) in Cx45fl/fl:Nestin-Cre mice but not in Cx45fl/fl:TIE2-Cre (C, gracilis artery marked with arrows) and Cx45fl/fl controls (D: gracilis artery, G, H: femoral artery). The vein (lumen marked by *) accompanying the gracilis artery was not stained in Cx45fl/fl:Nestin-Cre (A) or Cx45fl/fl:TIE2-Cre (C) compared to control (D). Nuclei are stained in blue (TO-PRO-3). Images are representative for at least n = 3 each genotype. Calibration bars are 100 (A, C, D, E, G) or 50 µm (B, F, H).

Vascular smooth muscle cells were also strongly labelled with EGFP in Cx45fl/fl:Nestin-Cre mice in larger vessels including the gracilis artery which feeds a skeletal muscle (Fig. 2A, 2B) including its branches. Note, that expression in the accompanying vein was not observed (Fig. 2A). Likewise, small arteries within the mesenteric vascular bed were stained in these mice (not shown). In contrast, Cx45fl/fl:TIE2-Cre mice did not exhibit staining in the gracilis artery (Fig. 2C) or mesenteric vessels (not shown). These vessels were also devoid of EGFP staining in control mice (Cx45fl/fl) (Fig. 2D). In conducting vessels, EGFP staining was pronounced in smooth muscle in the femoral artery, but not in control mice (Fig. 2E to 2H). In other conducting vessels (carotid artery, aorta), EGFP staining was not unequivocally different from control mice (in part due to autofluorescence) suggesting that Cx45 expression is less pronounced in these conducting arteries.

**Figure 3:**
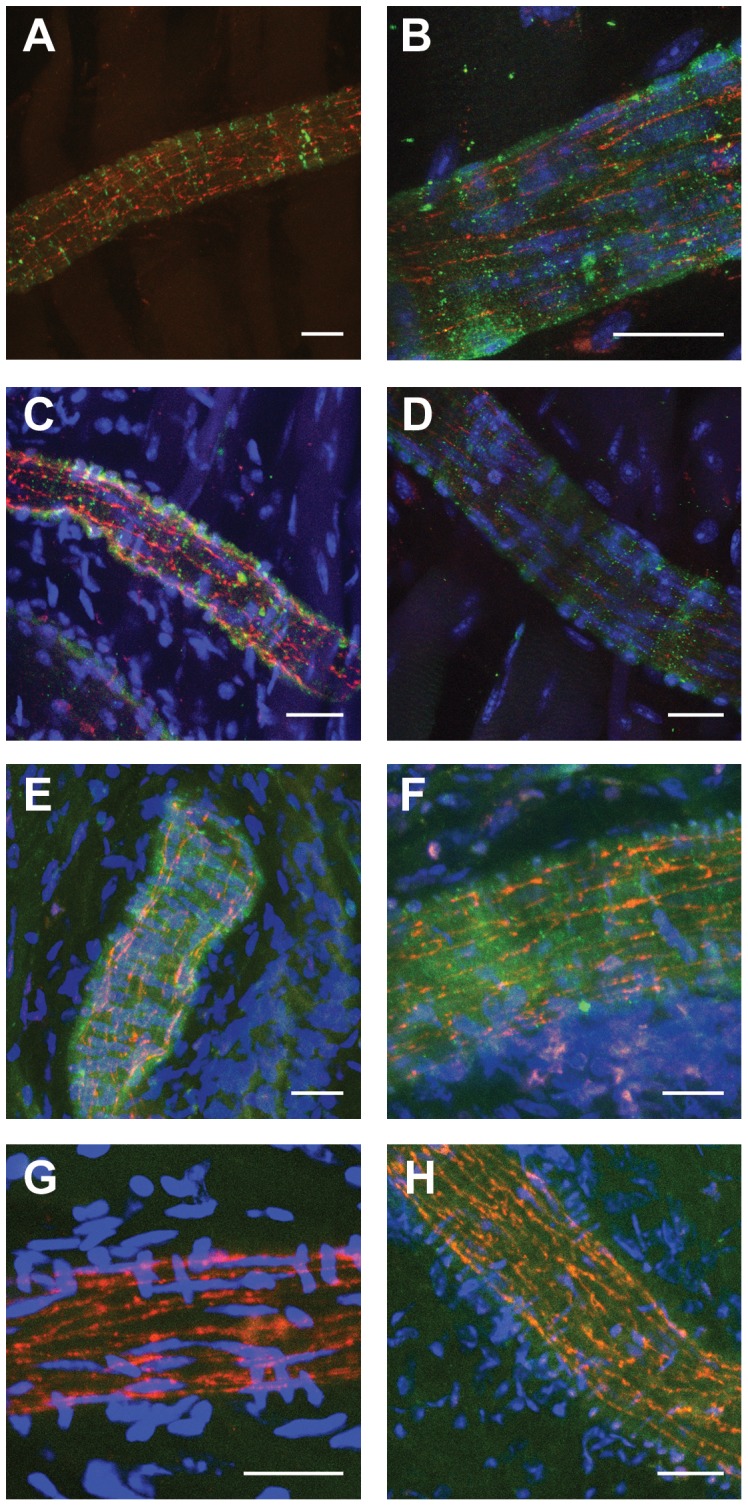
Connexin45 is not colocalized with endothelial connexins. In animals expressing a transgenic Cx45 tagged with EGFP, expression of Cx45 in arteriolar smooth muscle was confirmed. EGFP was visualized using an EGFP-targeted antibody (green) in whole-mount preparations of the microcirculation (cremaster muscle). EGFP staining is localized to membranes of cells wrapped circularly around the arterioles indicating Cx45 localisation in smooth muscle cell membranes (A–F). Additionally, expression and localisation of Cx37 (red, left panels) and Cx40 (red, right panels) was visualized using antibodies directed against these connexins. Red staining is observed in membranes of cells oriented along the length of the vessel which indicates that these are endothelial cells. Colocalisation of red and green stain is not found. In animals lacking EGFP-tagged Cx45, green staining is absent while red staining (G: Cx37, H: Cx40) is localized to endothelial cells. Images are representative for at least n = 3 each protocol or genotype. Calibration bars are 25 µm, nuclei are stained using TO-PRO-3 (blue).

The second model studied consisted of mice expressing a transgenic Cx45 tagged with the sequence for EGFP under the endogenous Cx45 promotor [30]. This allows to locate Cx45 within the cells without the need for a Cx45-targeted antibody. However, EGFP staining was too faint to be clearly identified and therefore EGFP-tagged Cx45 was visualized using an EGFP-targeted antibody. EGFP staining was localized along smooth muscle cell borders wrapped around the arterioles (Fig. 3). Co-staining using Cx37- and Cx40-targeted antibodies revealed that Cx37 (Fig. 3, left panels) and Cx40 (Fig. 3, right panels) are expressed in endothelial cells as shown before [21], [39], [40]. Interestingly, colocalisation of green and red stain was not found indicating that Cx45 is not expressed in the same cell as Cx40 or Cx37. In littermates, which lack EGFP-tagged Cx45, EGFP staining in arterioles was absent, while Cx37 or Cx40 was identified in endothelial cells (Fig. 3G, 3H).

### Basal microcirculatory data

A total of 41 arterioles were studied in 20 mice. Resting diameter of untreated arterioles (Cx45fl/fl: 20.5±2.2 µm, Cx45fl/fl:Nestin-Cre: 20.2±3.2 µm) and maximal diameter (Cx45fl/fl: 43.4±2.1 µm, Cx45fl/fl:Nestin-Cre: 38.1±2.5 µm) as measured during superfusion of sodium nitroprusside, adenosine, and acetylcholine (each 30 µmol/L) were not significantly different between genotypes. From these values the arteriolar resting tone was calculated as the fraction of resting from maximal diameter. The resting tone was also similar in both groups of mice (0.46±0.04 vs. 0.49±0.05, Cx45fl/fl and Cx45fl/fl:Nestin-Cre, respectively) indicating a similar contractile state of the arterioles at rest.

### Conducted vasoconstriction

**Figure 4:**
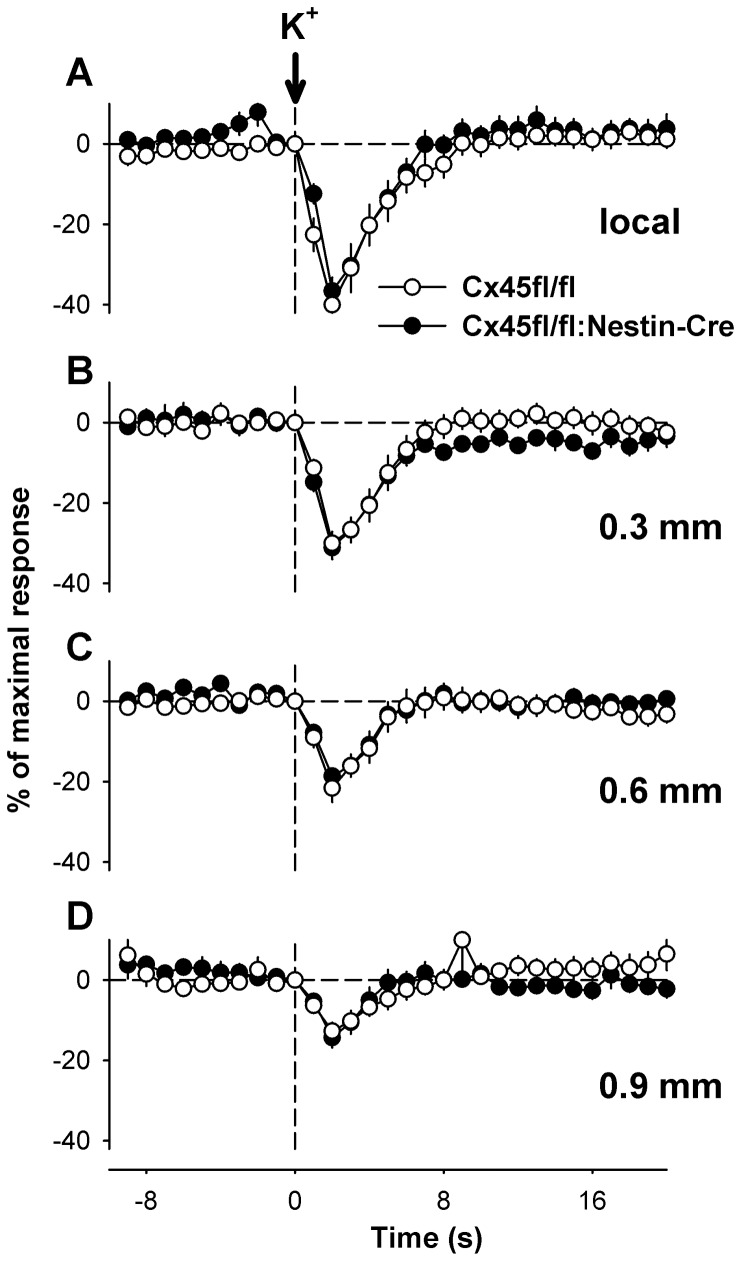
Conduction of KCl-induced constrictions in mice deficient for Cx45 in vascular smooth muscle. Time courses of local and upstream constrictions evoked by depolarizing K^+^ stimulation. Brief local application of K^+^ (3 mol/L) induced a rapid, transient constriction of the arterioles at the local site (A). The constriction conducted to remote, upstream sites (B: 0.3, C: 0.66, D: 0.9 mm) with a decreasing amplitude that was similar in Cx45fl/fl and Cx45fl/fl:Nestin-Cre mice. In both genotypes amplitudes decreased with increasing distance from the application site. Six or 8 arterioles were studied in 4 animals of each group.

**Figure 5:**
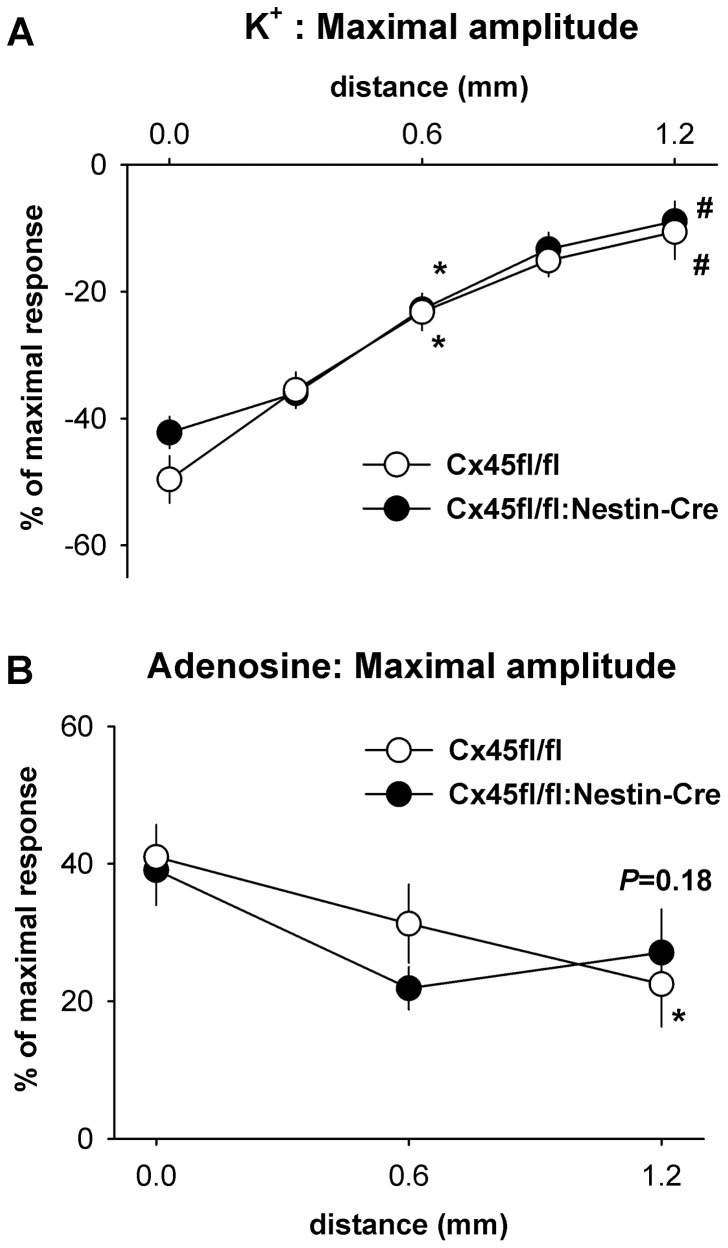
Conduction of vasomotor signals along the smooth muscle cell layer. Maximal amplitude of the constriction evoked by brief microapplication of depolarizing K^+^ solution (A) or application of adenosine (B, 10 mmol/L) are depicted as a function of distance from the stimulation site. A: The maximal amplitude of the K^+^-induced constriction decreased with distance and differences between genotypes were not observed (n = 6 or 8 arterioles in 4 animals each genotype). B: Likewise, the maximal amplitude of the adenosine-induced dilation decreased significantly with distance in Cx45fl/fl mice (n = 10 in 3 animals) and a similar trend was observed in less experiments in Cx45fl/fl:Nestin-Cre (n = 6 in 3 animals). Differences between genotypes were not observed. *: *P*<0.05 vs. local site, #: *P*<0.05 vs. 0.6 mm.

Conduction of vasomotor signals along the vessel was studied by local application of depolarizing K^+^ solution, the vasodilator adenosine, and the endothelium-dependent agonist acetylcholine (ACh). Responses to K^+^ and adenosine conduct most likely along the smooth muscle whereas ACh dilations conduct in a Cx40-dependent manner along the endothelial cell layer [25]. Brief local stimulation of the arterioles with a short pulse of depolarizing K^+^ solution (3 mol/L) induced a rapid constriction at the local site in control mice (Cx45fl/fl). A comparable local constriction was evoked by K^+^ stimulation in mice lacking Cx45 in vascular smooth muscle (Cx45fl/fl:Nestin-Cre, Fig. 4). The maximal amplitude (Fig. 5A), time to achieve this maximum, and the duration of the local response were not different between both genotypes. The local constriction was conducted without measurable time delay to upstream remote sites in control as well as Cx45fl/fl:Nestin-Cre mice (Fig. 4). However, the maximal amplitude decreased monotonically with distance (Fig. 5A). Most interestingly, conduction was unhindered in mice devoid of Cx45 in vascular smooth muscle and maximal amplitudes, time to achieve this maximum, as well as duration of the reponses were not different from responses obtained in floxed control animals (Fig. 4, 5A).

### Conducted vasodilation

Microapplication of the vasodilator adenosine (10 mmol/L) initiated a transient local dilation (response duration 43±4 s) with a maximal amplitude of 41±5% that was attained within 13±2 s in Cx45fl/fl mice. The amplitude and the temporal behaviour of the locally evoked response were similar in Cx45fl/fl:Nestin-Cre mice (Fig. 5B). In both genotypes, the dilation conducted to remote upstream sites with no measurable delay (not shown) but a decreasing amplitude (1.2 mm: Cx45fl/fl 23±6%, n = 10, *P*<0.05 vs. local; Cx45fl/fl:Nestin-Cre 27±6%, n = 6, *P* = 0.18 vs. local). Neither the amplitude nor the duration of the dilation in response to adenosine was different at any site in mice lacking Cx45 in vascular smooth muscle from the respective control animals. In a different experimental series, adenosine was examined after inhibition of nitric oxide synthase and cyclooxygenase (Nitro-*L*-arginine and indomethacin, 30 and 3 µmol/L). In this series, more vigorous stimulation using adenosine evoked a local dilation with a maximal amplitude of 81±6% in Cx45fl/fl and of 70±7% in Cx45fl/fl:Nestin-Cre mice (*P* = 0.25, n = 6 each). The dilation conducted to remote upstream sites and was not different in both genotypes at a distance of 1.2 mm (56±6 vs. 49±4%, Cx45fl/fl and Cx45fl/fl:Nestin-Cre mice, respectively; *P* = 0.60).

**Figure 6:**
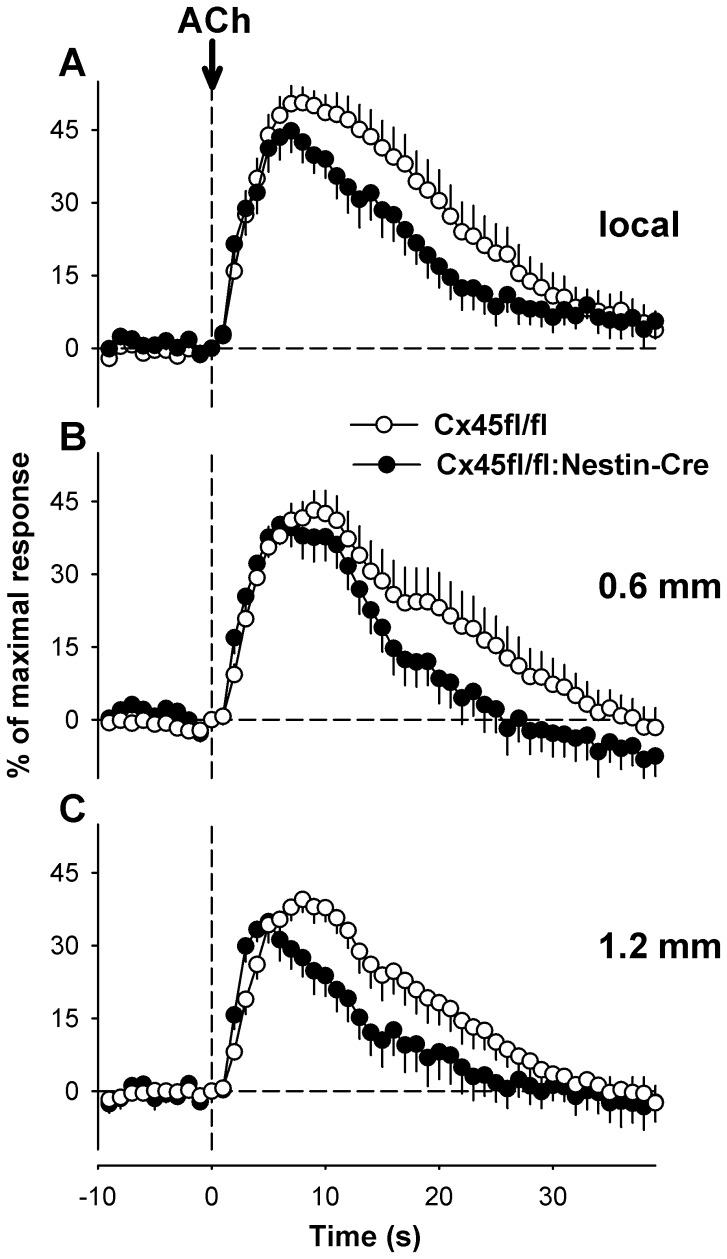
Conduction of endothelium-dependent dilations. Local and conducted dilations evoked by the endothelium-dependent agonist acetylcholine (ACh). Brief, local application of ACh (1 mmol/L) induced a transient local dilation (A) that was conducted to remote upstream sites (B: 0.6, C: 1.2 mm) without measurable delay in both genotypes. The maximal amplitude of the local dilation was achieved earlier and the dilation was shorter in Cx45fl/fl:Nestin-Cre animals reflecting most likely less vigourous stimulation. Nevertheless, the dilation conducted to remote sites up to the furthest distance studied also in these mice without attenuation of the amplitude. Six or 7 arterioles were studied in 4 or 5 animals of each genotype.

Additionally, the endothelium-dependent dilator ACh was studied. Local stimulation of the arterioles using ACh evoked a local dilation with a maximal amplitude of 58±3% that was attained within 11±1 s and lasted for 40±4 s in Cx45fl/fl mice which was conducted to remote sites with nearly unaltered amplitude (Fig. 6). The maximal amplitude of the local dilation was slightly lower in Cx45fl/fl:Nestin-Cre mice (47±4%, *P* = 0.05 vs. control) as well as the time to this maximum (8±1 s, *P*<0.05) and the response duration (30±3 s, *P*<0.05) which is most likely due to a less efficient stimulation. However, the conduction of the dilation to remote sites was not different in mice devoid of Cx45 in vascular smooth muscle from the respective control animals as verified by a similar maximal amplitude of the dilation at these sites (0.6 mm: 52±4% vs. 46±4%, *P* = 0.29; 1.2 mm: 46±3% vs. 43±2%, *P* = 0.47, Cx45fl/fl and Cx45fl/fl:Nestin-Cre, respectively). The temporal behaviour is depicted in figure 6.

### Arterial pressure

**Figure 7:**
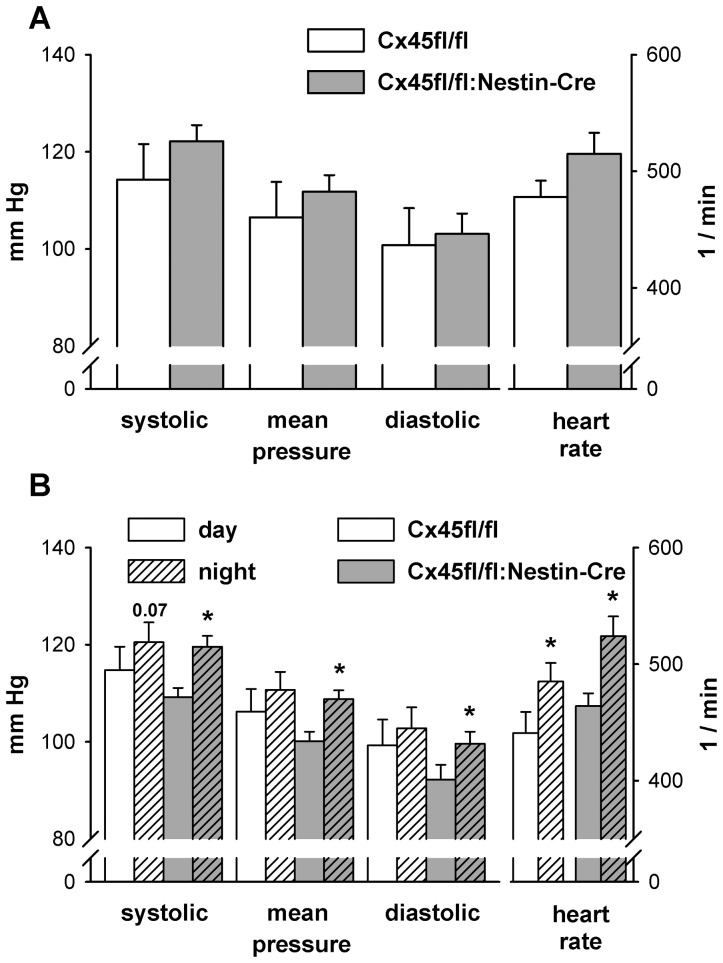
Arterial blood pressure in mice deficient for Cx45 in vascular smooth muscle. Arterial pressure measured by telemetry was not different between genotypes in conscious freely moving mice. A: Systolic, mean, and diastolic pressures as well as heart rate averaged for 24 h from measurements at day 5 to 7 after implantation of the telemetric device in Cx45fl/fl (white) and Cx45fl/fl:Nestin-Cre mice (grey) were not different from each other. B: Values obtained 12 to 14 days after implantation are separately depicted for day (plain) and night (hatched) indicating circadian rhythm which was pronounced in Cx45fl/fl:Nestin-Cre. Also at this time point pressures and heart rate were not different between genotypes. n = 8 animals each genotype, * *P*<0.05 vs. day values.

Arterial pressure was measured by telemetry in awake, freely moving mice in 8 animals of each genotype which were of a similar age (385±26 vs. 381±26 days, Cx45fl/fl and Cx45fl/fl:Nestin-Cre, respectively). Six days after implantation arterial pressure and heart rate were not different between the genotypes (Fig. 7A). After another week, circadian rhythm was established as verified by higher values for pressure and heart rate during night time when mice are more active which was pronounced in Cx45fl/fl:Nestin-Cre mice. However, also at this time point differences between genotypes were neither observed for systolic, mean, or diastolic pressures nor for heart rate during day or night (Fig. 7B).

## Discussion

The present study demonstrates that Cx45 is expressed in smooth muscle cells of arterioles and larger conducting vessels (A. gracilis, femoral, and mesenteric arteries) whereas Cx45 seems to be not expressed in the largest arteries (carotid artery and aorta). Moreover, smooth muscle cells in venules and pericytes surrounding capillaries exhibit Cx45 promotor activity, but endothelial cells of arterioles and venules lack this activity. Although immunolabelling cannot verify colocalisation of different Cx in gap junction plaques, our data do not provide any evidence of colocalisation of Cx45 with endothelial connexins, namely Cx37 and Cx40. Despite convincing evidence for the expression of Cx45 in smooth muscle cells in arterioles we were unable to identify a functional phenotype related to smooth muscle specific deletion of Cx45. Neither the conduction of vasomotor responses along the arteriolar wall in the microcirculation nor the arterial pressure in freely moving conscious mice were altered which suggests that Cx45 is not a crucial gap junctional component in vascular smooth muscle cells and in the regulation of arterial pressure.

In contrast to Cx37, Cx40, and Cx43, the expression of Cx45 in the vascular system has been addressed only in a limited number of studies mostly in the rat. Its expression was demonstrated by immunostaining and western blotting in smooth muscle cells of small and larger arteries in the cerebral circulation of the rat [41], in murine renal arterioles [42]–[44], and additionally in some large conducting vessels in the rat, such as the aorta, the femoral artery, or the caudal artery [45], [46]. In most cases expression levels of Cx45 were reported to be low in comparison to Cx43 [45], restricted to certain areas of the aorta (i.e. the ascending aorta) [46], or even absent in the media of cerebral vessels [47]. These observations rely on the specificity and sensitivity of the Cx45 antibodies used. A distinct approach is the targeted replacement of Cx45 by a reporter gene which verifies the transcriptional expression of Cx45. In mice, which carried such an exchange ubiquitously in a heterozygous fashion, the reporter gene *lacZ* was expressed in visceral as well as in vascular smooth muscle, for example in large conducting arteries (aorta) and the renal circulation [27]. The ubiquitous deletion of Cx45 is embryonically lethal which precludes analysing Cx45 function in the adult animal using this approach [27], [28]. Therefore, we used in the present experiments animals in which Cx45 was deleted specifically in smooth muscle using Cre-recombinase controlled by the Nestin promoter. In these animals, the deletion of Cx45 is also accompanied by the expression of a reporter protein (EGFP) [31], [32] and thus EGFP detection verifies trancriptional expression of Cx45 as well as successful deletion of Cx45. In fact, we detected EGFP in smooth muscle cells of various arterial vessels, i.e. in the mesenteric circulation, in large conducting (A. femoralis) as well as small arteries (A. gracilis) and also in arterioles within the microcirculation feeding skeletal muscle tissue (Fig. 1, 2). In addition to arterioles, EGFP was also expressed in venular smooth muscle cells and cells at the capillary level, most likely pericytes. However, larger veins (gracilis vein with a diameter of ∼100 µm) were devoid of EGFP staining. In contrast to smooth muscle cells, transcriptional activity of Cx45 was not detected in endothelial cells. This transcriptional activity only in arteriolar smooth muscle is in line with faint Cx45 immunostaining detected in this preparation previously [39]. However, Cx45 was not detected by immunostaining in cells in venules or capillaries. The cellular localisation of Cx45 was further and also independently of Cx45 antibodies studied in mice expressing an EGFP-tagged Cx45 fusion protein by means of a bacterial artificial chromosome which contained the Cx45 gene and regions more than 50 kb up- and downstream thereof. As shown previously, this fusion protein mimics Cx45 expression in wildtype mice in cardiac cells and bronchioles [30], [48]. Although the EGFP fluorescence was too faint to be detected by confocal microscopy in the microcirculation the localisation of the fusion protein could be traced using an EGFP-targeted antibody which excludes possible antibody cross-reactivity with other connexins. Thus, we detected the Cx45 fusion protein in the plasma membrane of smooth muscle cells in arterioles but not in venules or pericytes. A colocalisation with connexins expressed in endothelial cells (Cx40 or Cx37) was not observed in arterioles, which argues against a role of Cx45 in myoendothelial gap junctions. Taken together, we provide evidence for the expression of Cx45 and its localisation in the plasma membrane in smooth muscle cells of arterioles and arteries preferentially in smaller vessels. Transcriptional activity of Cx45 was also observed in venules and possibly pericytes which, however, does not necessarily entail expression of the protein and its localisation in the cell membrane.

Our functional experiments failed to document a crucial role for Cx45 in the conduction of locally initiated vasomotor responses along arterioles in the microcirculation. The vascular wall comprises two distinct pathways along which signals are conducted depending on the cell type that is initially engaged by the vasoactive substance [25]. Adenosine dilates murine arterioles most likely independently of the endothelium by the activation of ATP-dependent K^+^-channels in smooth muscle cells [25]. Consequently, the conduction of adenosine-induced dilations are thought to travel along homocellularly coupled smooth muscle cells. However, the conduction of these dilations remained fully intact in animals devoid of Cx45 in smooth muscle cells. Similarly, the conduction of constrictions initiated by local application of high K^+^-solution [49] was unaltered in SMC-specific Cx45 deficient mice. This suggests that Cx45 is dispensable for conduction of vasoactive signals along the smooth muscle cell layer. Because the endothelium-dependent dilator acetylcholine (ACh) primarily hyperpolarizes endothelial cells through activation of Ca^2+^-dependent K^+^-channels (K_Ca_3.1) [36], [50] the dilatory signal conducts also along this strongly coupled layer in a Cx40-dependent fashion [21], [39]. Therefore, the integrity of the conduction of ACh-induced dilations in SMC-specific Cx45-deficient mice was expected. The direct transfer of the ACh-induced endothelial hyperpolarization through myoendothelial gap junctions to the adjacent smooth muscle cell is in arterioles *in vivo* not the main dilatory mechanism [3], [40], [51]. The intact dilation in response to ACh application in SMC-specific Cx45-deficient mice is in line with this view as is the finding that Cx45 does not colocalize with Cx40 and Cx37 expressed in endothelial cells. Summarized, we did not find a functional phenotype related to Cx45 deficiency in smooth muscle cells. Currently, only electrophysiologic measurements of the coupling of vascular smooth muscle isolated from the cerebral circulation suggested that functional gap junction channels are formed by Cx45 in rats [52].

Previously, Hanner and coworkers reported an elevated arterial pressure in anesthetized mice deficient for Cx45 in smooth muscle cells which was associated with enhanced plasma renin levels [29]. Our data demonstrate that arterial pressure in non-anesthetized, conscious mice was not different between controls and animals lacking Cx45 in smooth muscle cells. After about 12 days of continuous telemetric measurement the animals reestablished a circadian rhythm with higher pressure and heart rate at night during which the animals are more active and also at this time point of the experiment systolic and diastolic arterial pressures were not different during day or night between the genotypes. These measurements convincingly exclude an elevated pressure in conscious, freely moving SMC-specific Cx45-deficient mice. The contrasting results to the previous study are not due to a different strategy to target chromosomal recombination in smooth muscle which was in fact similar (Nestin promoter driven Cre-recombinase) but they are most likely related to the anesthetic protocol used by Hanner and coworkers because the depth of anaesthesia easily affects arterial pressure in conjunction with the limited number of experiments performed in that study (n = 3) [29]. In addition, we used mice homozygously carrying the floxed Cx45 gene originating from the same breeding colony as controls whereas in that previous study C57BL/6 were taken as control mice. These authors also suggested that Cx45 is expressed in renin-producing cells which was, however, not found in a very recent study using analysis of *lacZ* expression controlled by the Cx45 promoter [53] or using immunostaining [44]. In addition, the deletion of potentially expressed Cx45 in renin-producing cells using a Cre-recombinase controlled by the renin promoter did not produce an elevation of arterial pressure or enhanced plasma renin activity at rest or after challenge using salt depletion [53]. In summary, Cx45 does not seem to be crucially involved in the regulation of blood pressure. It remains to be determined if Cx45 expressed in smooth muscle cells in fact alters renin secretion, while the role of Cx45 in renin-secreting cells itself seems to be not of major importance.

The global deletion of Cx45 and its lethality was associated with vascular malformations [27] which suggested a role for Cx45-dependent gap junctional coupling in vascular cells to enable the maturation of blood vessels. However, mice lacking Cx45 in smooth muscle cells (Nestin promoter driven Cre-recombinase) or carrying a deletion of Cx45 in endothelial cells (TIE-2 promoter driven Cre-recombinase) were viable. We did not study embryonic lethality in detail because we obtained animals with EC- or SMC-specific Cx45 deletion which argues against a crucial role of Cx45 in either cell type alone for the vessel maturation. Interestingly, cardiac-myocyte specific Cx45 deletion resulted in embryonic lethality [54] and was associated with a similar phenotype as reported in the germline deficient mice by these investigators [28]. Thus, the generation of cell-specific deficient animals in conjunction with the expression of reporter genes is a powerful technique to foster our knowledge of connexin physiology [55]. Our study suggests that the function of Cx45 in smooth muscle cells can be replaced by other connexins and/or that the approaches are currently not subtle enough to dismantle their function in detail. Unfortunately, despite clear evidence of Cx45 expression in smooth muscle cells its function remains to be uncovered.
